# The NO/cGMP pathway inhibits transient cAMP signals through the activation of PDE2 in striatal neurons

**DOI:** 10.3389/fncel.2013.00211

**Published:** 2013-11-18

**Authors:** Marina Polito, Jeffrey Klarenbeek, Kees Jalink, Danièle Paupardin-Tritsch, Pierre Vincent, Liliana R.V. Castro

**Affiliations:** ^1^UMR7102, Centre National de la Recherche ScientifiqueParis, France; ^2^UMR7102, Neurobiology of Adaptive Processes, Université Pierre et Marie CurieParis, France; ^3^Cellbiophysics Group, The Netherlands Cancer InstituteAmsterdam, Netherlands

**Keywords:** cyclic AMP, cyclic GMP, phosphodiesterase, biosensor imaging, nitric oxide, striatum, dopamine

## Abstract

The NO-cGMP signaling plays an important role in the regulation of striatal function although the mechanisms of action of cGMP specifically in medium spiny neurons (MSNs) remain unclear. Using genetically encoded fluorescent biosensors, including a novel Epac-based sensor (EPAC-S^H150^) with increased sensitivity for cAMP, we analyze the cGMP response to NO and whether it affected cAMP/PKA signaling in MSNs. The Cygnet2 sensor for cGMP reported large responses to NO donors in both striatonigral and striatopallidal MSNs, this cGMP signal was controlled partially by PDE2. At the level of cAMP brief forskolin stimulations produced transient cAMP signals which differed between D_1_ and D_2_ MSNs. NO inhibited these cAMP transients through cGMP-dependent PDE2 activation, an effect that was translated and magnified downstream of cAMP, at the level of PKA. PDE2 thus appears as a critical effector of NO which modulates the post-synaptic response of MSNs to dopaminergic transmission.

## Introduction

Cyclic nucleotides control a range of cellular processes, particularly in neurons where they transduce extracellular signals carried by neuromodulators. The striatum is involved in reward, motor control and action selection, and cyclic nucleotide signaling plays a critical role in the normal function of this brain structure. While the involvement of cAMP in striatal physiology is widely acknowledged, much less is known on cGMP, although this signaling cascade also plays a critical role in the regulation of striatal function (West and Tseng, 2011). Medium spiny neurons (MSNs) in the striatum constitute 95% of the neuronal types, approximately half of which are anatomically defined as the “direct pathway” and express dopamine type 1 receptors (D_1_). This receptor is positively coupled to cAMP production. The other half of MSNs, defined as the “indirect pathway,” express high levels of dopamine type 2 receptors (D_2_) and adenosine A_2A_ receptors (Le Moine and Bloch, 1995; Bateup et al., 2008; Bertran-Gonzalez et al., 2008). D_2_ receptors are negatively coupled to adenylyl cyclases (AC) and therefore inhibit cAMP production while the A_2A_ receptors are positively coupled to AC and increase cAMP levels. The segregation between D_1_ and A_2A_ expressing neurons is clearly visualized using cAMP/PKA biosensors (Castro et al., 2013). The functional effects of the cAMP signaling cascade has been widely documented in the striatum (Hervé and Girault, 2005) but much less is know about the cGMP signaling. cGMP is produced by the NO receptor, aka soluble guanylyl cyclase (sGC), in response to nitric oxide (NO), and NO/cGMP signaling regulates a number of neurobiological processes (Garthwaite, 2008). sGC is highly expressed in the striatum (Ariano et al., 1982; Matsuoka et al., 1992; Ding et al., 2004) while the NO producing enzyme nNOS is highly expressed by a fraction of striatal interneurons (Vincent and Kimura, 1992; Rushlow et al., 1995; Kawaguchi, 1997; Vincent, 2000). It is commonly accepted that NO produced by NOS interneurons diffuses throughout the striatal complex and increases corticostriatal and dopaminergic synaptic transmission via a sGC-cGMP dependent mechanism (West et al., 2002; West and Grace, 2004; West and Tseng, 2011). In addition, NO diffuses into the dendrites of MSNs modulating corticostriatal synaptic plasticity *in vitro* (Calabresi et al., 1999, 2000) and *in vivo* (West and Grace, 2004). While these effects are clearly mediated by cGMP, the downstream effectors of cGMP, often assumed to be the cGMP-dependent protein kinase (PKG), remain uncertain, since only moderate levels of expression have been reported in the striatum (el-Husseini et al., 1995; El-Husseini et al., 1999; de Vente et al., 2001) and CNG expression has not been reported in the striatum (Wei et al., 1998).

An important part of the signal transduction process is the rapid degradation of the cyclic nucleotides by cyclic nucleotide phosphodiesterases (PDEs). PDE2 mRNA and protein are present at high levels in the striatum (Repaske et al., 1993; Van Staveren et al., 2003) and functionally active (Wykes et al., 2002; Lin et al., 2010). PDE2 has dual enzymatic activity allowing to hydrolyze both cAMP and cGMP (Erneux et al., 1981; Martins et al., 1982). A characteristic feature of PDE2 is the positive cooperativity of the substrate cGMP: in the absence of cGMP, PDE2 activity is low, and the binding of sub-micromolar cGMP to the regulatory GAF-B domain of the amino-terminus of PDE2 results in a 5-fold increase in cAMP hydrolysis rate (Martins et al., 1982; Martinez et al., 2002). The interplay between cAMP and cGMP signals through PDE2 has been well characterized mainly in the cardiovascular system (Maurice, 2005; Nikolaev et al., 2005), where cGMP-mediated regulation of cAMP occurs in a spatially confined cellular compartment and depends on the source of cGMP (Castro et al., 2006; Stangherlin et al., 2011). Regulation of cyclic nucleotides by PDE2 has already been shown in thalamic (Hepp et al., 2007) and striatal neurons (Lin et al., 2010), although in the striatum the role of PDE2 at the cellular level remained to be analyzed. Here, we improved the ^T^Epac^*VV*^ cAMP sensor (Klarenbeek et al., 2011) which allowed us to analyze the dynamics of cAMP regulation in striatal neurons and to determine the functional effect of cGMP on this signal.

Our data reveal that NO/cGMP signaling reduces the cAMP signals in both striatonigral and striatopallidal MSNs through the activation of PDE2. This inhibitory effect propagates downstream to PKA, leading to an inhibition of the PKA response to D_1_ stimulation. In addition, we found that the dynamics of the cAMP responses are not identical in MSNs, with striatopallidal neurons displaying larger and longer lasting cAMP transients than striatonigral MSNs.

## Materials and methods

### Biosensor construct and validation

From the starting material TEpacVV (Klarenbeek et al., 2011), numbered Epac-SH74 in our database, we first exchanged mTurquoise for mTurqouise2 using the same protocol as in Klarenbeek et al., creating Epac-SH126. Second the Q270E mutation was introduced by cutting the Epac-SH126 with PshAI en BstEII inserting annealed oligo's forward primer: GTGACCCATGGCAAGGGGCTGGTGACCACCCTGCATGAGGGAGATGATTTTGGAGAGCTGGCTCTGGTCAATGATGCACCCCGGGCAGCCACCATCATCCTGCGAGAAGACAA and reverse primer: TTGTCTTCTCGCAGGATGATGGTGGCTGCCCGGGGTGCATCATTGACCAGAGCCAGCTCTCCAAAATCATCTCCCTCATGCAGGGTGGTCACCAGCCCCTTGCCATGG, yielding Epac-SH134. Third the acceptor was inserted as a PCR-product of cp174Citrine using forward primer: GGGGCTAGCGAGCTCATGGACGGCGGCGTGCA and reverse primer: CGAATTCGGCTCGATGTTGTGGCGGAT digested with NheI and EcoRI, yielding Epac-SH150. All constructs were checked by sequence analysis. Hek293 embryonal kidney cells (American Type Culture Collection crl-1573) were cultured in DMEM supplemented with 10% FCS and antibiotics. Cells were seeded in six 15 cm^2^ plates and transfected with 10 μg DNA per plate using calcium phosphate or fugene transfection agent. After overnight expression, cells were resuspended in 1 ml hypotonic buffer (PBS: H_2_O 1: 2) and homogenized with a Downs piston. The homogenate was centrifuged for 10 min at 4°. The supernatant was corrected toward isotonic conditions using a concentrated stock of PBS. The supernatant was diluted 10 times in buffer containing (in mM) 150 KCl, 5 NaCl, 1 MgCl_2_ 10 HEPES pH 7.2, with a total volume of 2 ml in a stirred cuvette of a PTI Quantamaster dual channel spectrofluorimeter (Lawrenceville, NJ). Small volumes of cAMP from concentrated stocks were added repeatedly to titrate in cAMP, total added volume 40 μl. The response to cAMP was quantified as the ratio between YFP (530 ± 10 nm) and CFP (490 ± 10 nm), when excited with 420 ± 3 nm.

### Brain slice preparation

Wild-type C57Bl/6J mice were obtained from Janvier (Le Genest Saint Isle, France). Mice were maintained in a 12 h light–12 h dark cycle, in stable conditions of temperature (22°C), with food and water available *ad libitum*. All the experiments were performed according to French Ministry of Agriculture and Forestry guidelines for handling animals (87–848°).

Brain slices were prepared from male mice aged from 9 to 13 days, as previously described (Castro et al., 2013). Coronal brain slices of 300 μm thickness were cut with a VT1200S microtome (Leica, Germany). Slices were prepared in an ice-cold solution of the following composition: 125 mM NaCl, 0.4 mM CaCl_2_, 1 mM MgCl_2_, 1.25 mM NaH_2_PO_4_, 26 mM NaHCO_3_, 25 mM glucose and 1 mM kynurenic acid, saturated with 5% CO_2_ and 95% O_2_. The slices were incubated in this solution for 30 min and then placed on a Millicell-CM membrane (Millipore) in culture medium (50% Minimum Essential Medium, 50% Hanks' Balanced Salt Solution, 6.5 g/l glucose, penicillin-streptomycin, Invitrogen). We used the Sindbis virus as a vector to induce expression of the various probes (Ehrengruber et al., 1999).

The sindbis viral vector for AKAR3 and Cygnet2 was prepared as as previously described (Gervasi et al., 2007; Hepp et al., 2007). Similarly, the Epac-S^H150^ digested with HindIII was inserted into pSinRep5 (Invitrogen, San Diego, CA) digested with StuI and made blunt by Klenow and HpaI.

Compared to our previous work (Castro et al., 2013), the viral vector was diluted to decrease the number of infected neurons and thus facilitate individual cell measurement. Slices were incubated overnight at 35°C under an atmosphere containing 5% CO_2_. Before the experiment, slices were incubated for 30 min in the recording solution (identical to the solution used for cutting, except that the calcium concentration was 2 mM and kynurenic acid was omitted). During recordings, brain slices were continuously perfused with this solution saturated with 5% CO_2_/95% O_2_, at a rate of 2 ml/min, in a recording chamber of ~1 ml volume maintained at 32°C. The viability of the neurons in these experimental conditions have been checked by patch-clamp recording, which showed electrical activity to be normal (Gervasi et al., 2007; Castro et al., 2010).

### Optical recordings on brain slices

Recordings were made on MSNs, that constitute 95% of neurons in the striatum. Large neurons, presumably cholinergic interneurones, were excluded (i.e., diameter larger than 14 μm). Wide-field images were obtained with an Olympus BX50WI or BX51WI upright microscope with a 20 × 0.5 NA or a 40 × 0.8 NA water-immersion objective and an ORCA-AG camera (Hamamatsu). Images were acquired with iVision (Biovision, Exton, PA, USA). The excitation and dichroic filters were D436/20 and 455dcxt. Signals were acquired by alternating the emission filters, HQ480/40 for CFP, and D535/40 for YFP, with a filter wheel (Sutter Instruments, Novato, CA, USA). All filters were obtained from Chroma Technology (Brattleboro, VT, USA). Image acquisition was triggered manually, except for kinetics measurement where images were acquired automatically with 3–5 s intervals.

Images were analyzed with custom routines written in the IGOR Pro environment (Wavemetrics, Lake Oswego, OR, USA). The emission ratio was calculated for each pixel: F535/F480 for AKAR3 and F480/F535 for Epac-S^H150^. Pseudocolor images display the ratio value coded in hue and the fluorescence intensity coded in intensity. A calibration square indicates the intensity values from left to right and the ratio values from bottom to top. The size of the square indicates the scale of the image in microns. No correction for bleed-through or direct excitation of the acceptor was applied and the ratio changes in our conditions therefore appear smaller than those reported by other studies in which such corrections were applied.

### Fast drug application

A fast focal application system was previously used for kinetic studies (Gervasi et al., 2007; Castro et al., 2013). A glass pipette (100–150 μm tip diameter) was placed 300 μm to the side of and 200 μm above the brain slice and ejected the drug contained in the same solution as the bath. Dopamine uncaging was performed with UV light at 360 nm applied in wide-field mode (UVILED, Rapp OptoElectronic, Hamburg, Germany). Image acquisition in these fast wide-field recordings was automatic at a frequency ranging from 0.2 to 0.3 Hz. Image acquisition was otherwise triggered manually by the user.

### Data analysis and statistics

Ratiometric quantification was performed with a ratio value between the Rmin and Rmax values, which correspond to the minimal ratio value (no biological signal) and maximal response (saturated biosensor) (Grynkiewicz et al., 1985; Börner et al., 2011). The baseline ratio in control conditions was considered to be equal to Rmin because adenylyl cyclase inhibition with 50 μM SQ22536 and guanylyl cyclase inhibition with 10 μM ODQ yielded no ratio decrease with Cygnet2 biosensor. The maximal response (Rmax, corresponding to biosensor saturation) was determined for each neuron at the end of the recording. This level was determined by applying 13 μM forskolin (for cAMP) or SNAP (for cGMP) in the presence of the broad-spectrum phosphodiesterase inhibitor IBMX (200 μM). Absolute ratio values differed between cells [as shown with the mutant biosensor in Castro et al. (2013)], so the amplitude of the response to receptor stimulation was quantified for each neuron as the fractional change in ratio from its own baseline (Rmin) and maximal final ratio response (Rmax).

Measurements were performed on regions of interest and some of the signal measured on a region of interest comes from out-of-focus neurons. Regions of interest which displayed clear responses to both SKF38393 and CGS21680 and which therefore contained fluorescence signal from out of focus cells were discarded from our analysis.

Kinetic parameters (amplitude, *t*_max_ and *t*_1/2off_) were determined using IGOR Pro environment (Wavemetrics, Lake Oswego, OR, USA). *t*_max_ values were determined as the time to reach the peak of the response and the *t*_1/2off_ represents the time to reach a half of the recovery of the response.

We analyzed at least four neurons per brain slice, with n indicating the number of independent neurons tested. Unpaired two-tailed student's *t*-tests were used for statistical comparisons. Differences were considered significant when *P* < 0.001.

### Drugs

SKF38393 hydrobromide, CGS21680 hydrochloride, 3-isobutyl-1-methylxanthine (IBMX), rolipram, NPEC-caged dopamine [(N)-1-(2-nitrophenyl) ethylcarboxy-3, 4-dihydroxyphenethylamine], and forskolin were obtained from Tocris Cookson (Bristol, UK); 1H-[1,2,4]oxadiazolo[4,3-a]quinoxalin-1-one (ODQ), S-nitroso-N-acetyl-D,L-penicillamine (SNAP), diethylamine NO (DEANO), erythro-9-(2-hydroxy-3-nonyl)-adenine (EHNA), BAY-60–7550 were obtained from Sigma-Aldrich (St Quentin Fallavier, France); BAY60–7550 was obtained from Cayman (Teaduspargi, Estonia).

## Results

### NO activates the sGC/cGMP signaling cascade in MSNs

**Figure 1:**
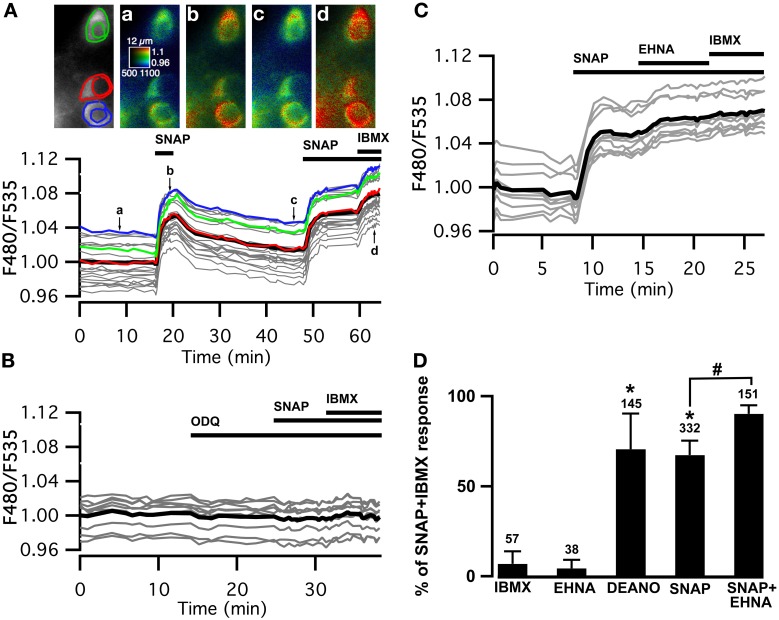
**Cygnet reports increases in cyclic GMP concentration in all medium spiny neurons of the striatum in response to soluble guanylyl cyclase activation. (A–C)** Striatal neurons in a mouse brain slice expressing Cygnet2 sensor and imaged by wide-field microscopy. **(A)** Images show the raw fluorescence at 535 nm (left in gray scale) and the ratio (in pseudocolor), indicating the ratiometric change of Cygnet2 reporting the binding of cGMP, at the times indicated by the corresponding arrows on the graph below. Each trace on the graph indicates the F480/F535 emission ratio measurement on regions indicated by the color contour drawn on the raw image; the thick black line represents the average of all the traces. The NO donor SNAP (100 μM) induced a strong increase in the ratio in all MSNs visible in the field. SNAP + IBMX (200 μM) induced a maximal increase in the ratio response. **(B)** The responses to SNAP and SNAP + IBMX were completely blocked by the soluble guanylyl cyclase blocker ODQ (10 μM). **(C)** After the activation of sGC with SNAP, the PDE2 inhibitor EHNA (10 μM) increased the cGMP signal to a level that corresponded to the maximal response. **(D)** The amplitude measured with the Cygnet2 biosensor was normalized with respect to the maximal SNAP + IBMX response and plotted as a histogram. Error bars indicate s.e.m.; the numbers of tested neurons are indicated above each bar. Unpaired two-tailed *t*-tests were carried out for comparisons with control conditions (^*^) and with SNAP alone (#), and differences were considered significant when *P* < 0.05.

We used the cGMP sensor Cygnet2 (Honda et al., 2001) to determine whether NO donors increase cGMP concentration in MSNs. The NO donor S-nitroso-N-acetyl-D,L-penicillamine (SNAP, 100 μM) induced a large increase in the F480/F535 emission ratio of all the cygnet-expressing MSNs (Figure 1A). This signal reversed with the washout of the drug and a second cGMP response could be elicited from the same cells, showing that the NO-cGMP signaling pathway can be activated repeatedly over the time-course of our recordings.

The steady-state cGMP level upon sGC stimulation depends on the relative activities of sGC and cGMP degradation mediated by PDEs. In the presence of the NO donor, the non-specific PDE inhibitor IBMX (200 μM) increased the ratio to a higher steady-state level, showing that PDE activities determine the steady-state cGMP level reached upon NO-mediated sGC stimulation. SNAP alone increased the emission ratio to 67 ± 8% (*n* = 332) of this maximal response. Similar responses (71 ± 20%; *n* = 145) were obtained with the NO donor diethylamine NO (DEANO, 100 μM). As expected, sGC inhibition by ODQ (10 μM) prevented the response to SNAP and SNAP plus IBMX (Figure 1B).

Previous studies performed with dissociated striatal neurons demonstrated that PDE2 regulates the cGMP responses to NO donors (Wykes et al., 2002; Lin et al., 2010). We tested the effect of PDE2 inhibition in our brain slice preparation and in all tested MSNs, the application of the PDE2 inhibitor EHNA (10 μM), added on top of the SNAP response, induced a further increase of the cGMP signal, rising from 67 ± 8% (*n* = 332) to 90 ± 5% (*n* = 151) of the maximal response obtained in the presence of IBMX (Figures 1C,D). Like IBMX, EHNA alone had no effect on basal cGMP levels.

These results confirm that PDE2 is critical in the regulation of the cGMP signals in MSNs of the dorsal striatum upon sGC stimulation.

### A new biosensor to measure cAMP signals in the striatum

**Figure 2:**
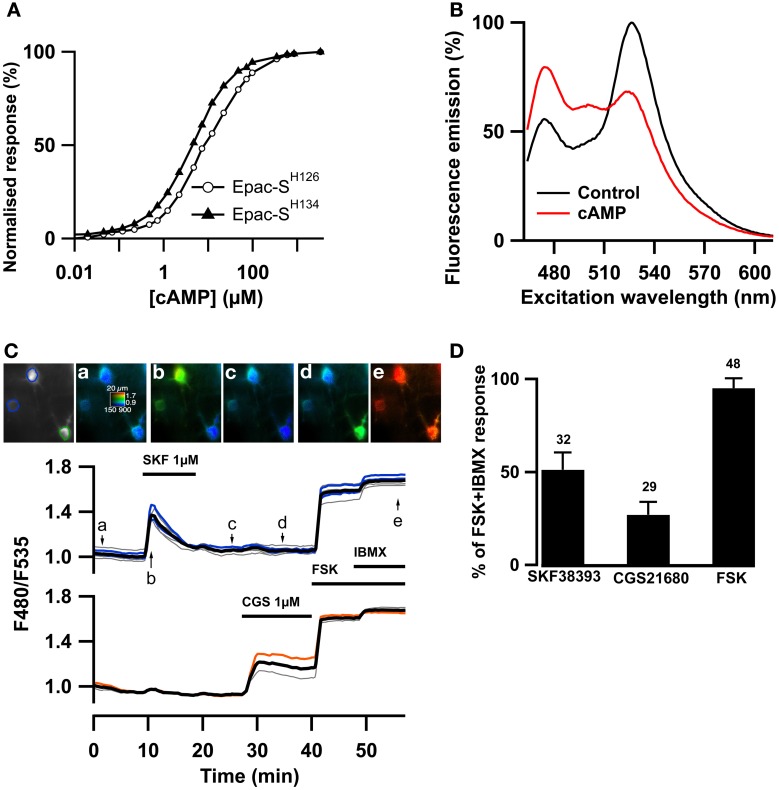
**The Epac-S^H150^ sensor reported large cAMP signals in D_1_ and D_2_ MSNs.** The ^T^Epac^VV^ sensor (labeled Epac-S^H126^) was mutated to increase its sensitivity for cAMP, yielding Epac-S^H134^; representative dose-response curves for both sensors are presented in **(A)**. The acceptor was then changed for a cp174Citrine, yielding Epac-S^H150^. The emission spectrum of Epac-S^H150^ in the absence and in the presence of saturating (100 μM) cAMP is presented in **(B)**. **(C)** Medium spiny neurons (MSNs) in a mouse brain slice were transfected for expression of Epac-S^H150^ and imaged with wide field microscopy. Images show the raw fluorescence at 535 nm (left, in gray scale) and the ratio (in pseudocolor) indicating the ratiometric change of Epac-S^H150^ reporting the binding of cAMP, at the times indicated by the arrows on the graph below. Each trace on the graph indicates the F480/F535 emission ratio measurement on regions indicated by the color contour drawn on the raw image; the thick black line represents the average of all the traces in the two different groups. The D_1_ receptor activator, SKF38393 (1 μM), induced a transient ratio increase in neurons thereby identified as D_1_ MSNs; activation of the A_2A_ receptor with CGS21680 (1 μM) induced a sustained cAMP response in neurons thereby identified as D_2_ MSNs. FSK + IBMX induced a ratio increase used as 100% value in normalization. **(D)** The amplitude of the responses to SKF38393 and CGS21680 was measured with the Epac-S^H150^ biosensor, normalized with respect to the maximal FSK + IBMX response and plotted as a histogram. The cAMP response to D_1_ receptor stimulation (SKF38393) was significantly stronger than the response to D_2_ receptor stimulation. Error bars indicate the s.e.m.; the number of tested neurons is indicated above each bar.

Since cGMP increases PDE2 activity which also hydrolyzes cAMP, we wanted to precisely monitor whether PDE2 controlled cAMP levels in striatopallidal and striatonigral MSN. The recently published ^T^Epac^VV^ (Epac-S^H126^) exhibits one of the largest ratio changes known to date for a genetically-encoded biosensors (Klarenbeek et al., 2011) but we considered that its relatively low sensitivity for cAMP may be limiting. We have prepared a series of new Epac-based cAMP biosensors in which the donor was replaced by mTurquoise2, which has a higher quantum yield, longer lifetime and is more photo stable than mTurquoise (Goedhart et al., 2012). The Q270E mutation (Dao et al., 2006) was introduced in the cAMP binding site of the cAMP-binding domain of Epac1 to increase its affinity for cAMP. This sensor, called Epac-S^H134^ showed increased sensitivity to cAMP as compared to the previous version (EC_50_ was 4.4 ± 0.3 vs. 10.7 ± 0.8 μM, *n* = 5, *p* < 0.01, two-tailed paired *t*-test; Figure 2A). This sensor was further improved by replacing the acceptor cpVenus-Venus with a single circular permutation of Citrine (cp174Citrine), chosen for optimal resistance to pH changes and brightness. This sensor called Epac-S^H150^ showed a large change in emission spectrum upon cAMP binding (Figure 2B) and proved suitable to directly address the dynamics of cAMP in MSNs in the striatum (Figure 2C). A detailed characterization of this and other new cAMP sensors will be published elsewhere.

As shown previously with PKA and cAMP biosensors with two-photon microscopy (Castro et al., 2013), activation of the D_1_ receptors with a saturating (1 μM) dose of SKF38393 strongly increased the F480/F535 emission ratio in one population of MSNs called hereafter D_1_ neurons. In addition, the activation of the A_2A_ receptors with a saturating (1 μM) dose of CGS21680 increased the emission ratio in the remaining neuronal population, the D_2_ neurons (Figure 2C). With low viral infection levels, wide-field imaging thus allowed a sufficient cell separation to distinguish between the D_1_ and D_2_ neurons. The D_1_ response decreased with prolonged exposure to the agonist, probably a consequence of D_1_ receptor desensitization. In contrast, the response to CGS21680 reached a stable steady-state level. Both responses were not maximal, as the addition of the adenylyl cyclase activator forskolin (FSK, 13 μM) further increased the ratio response, consistent with the wide cAMP sensitivity range of this new biosensor. Addition of 200 μM IBMX to forskolin produced a small additional response, considered as the maximal ratio response *R*_max_ (see methods). On average, SKF38393, CGS21680, and FSK increased the emission ration to 51 ± 2 % (*n* = 32), 27 ± 1 % (*n* = 29) and 95 ± 1% (*n* = 48) of the maximal response to forskolin and IBMX (Figure 2D).

### Brief adenylyl cyclase activation in D_1_ and D_2_ neurons

**Figure 3:**
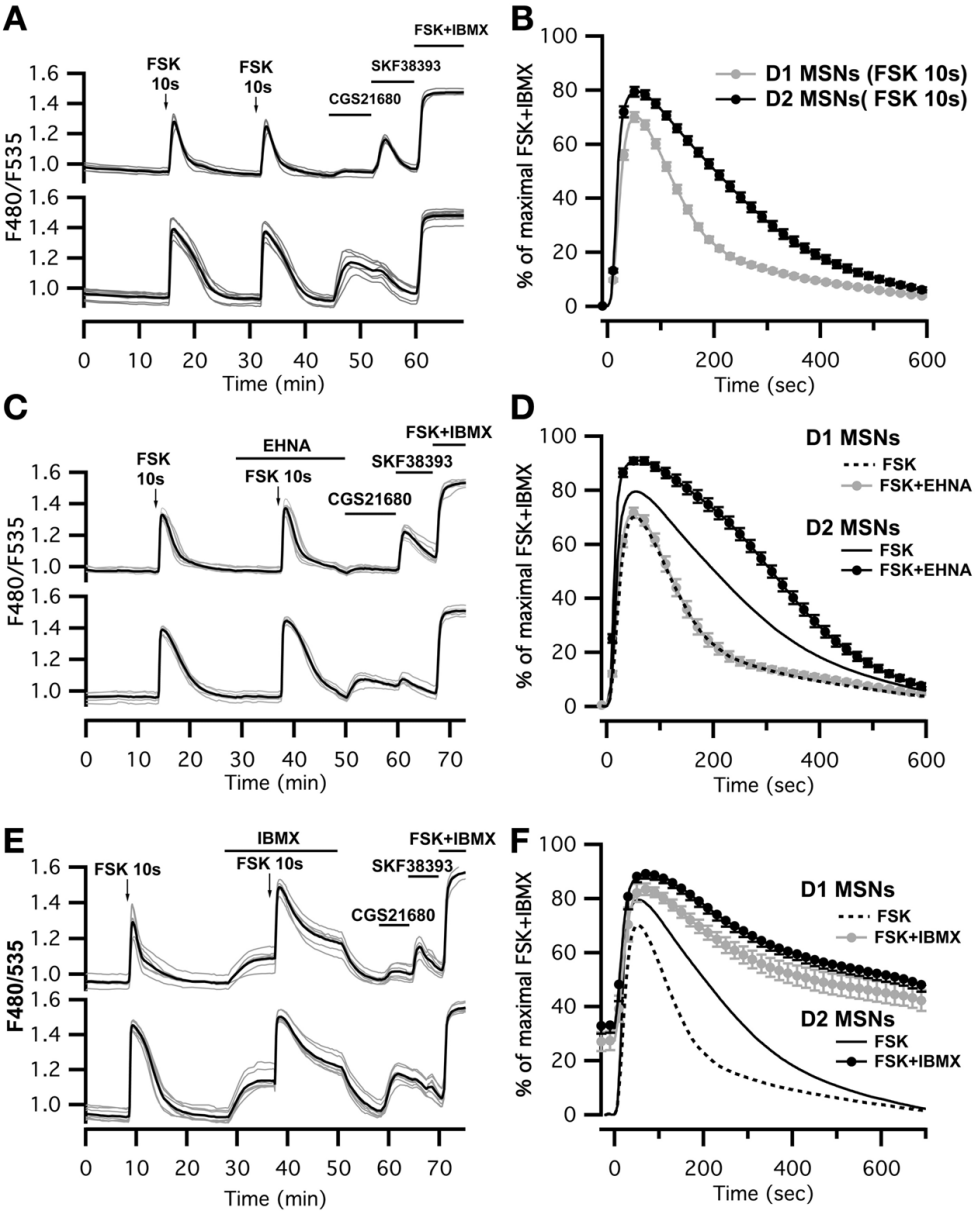
**Brief (10 s) application of FSK (13 μM) produced stronger and longer lasting cAMP responses in D_2_ MSNs than in D_1_ MSNs. (A,C,E)** cAMP signals were measured in wide-field imaging with Epac-S^H150^ biosensor during the application of a 10 s pulse of FSK alone **(A)**, in the presence of EHNA **(C)** or IBMX **(E)**. Each trace on the graph indicate the Epac-S^H150^ emission ratio of an individual neuron. Traces were separated on the basis of their response to the A_2A_ agonist CGS21680 (1 μM) or D_1_ agonist SKF38393 (1 μM). **(B)** Mean responses in D_1_ (*n* = 112) and D_2_ (*n* = 122) MSNs to brief FSK stimulation. **(D,F)** Mean responses in D_1_ and D_2_ to brief FSK stimulation in the presence of EHNA (10 μM) or IBMX (200 μM). Thin dashed and solid lines represent the mean responses in D_1_ and D_2_ MSNs to brief FSK alone, for comparison. IBMX strongly attenuated the differences between D_1_ (*n* = 17) and D_2_ (*n* = 21) MSNs. EHNA increased the cAMP signaling in D_2_ MSNs (*n* = 19), but had no effect in D_1_ cells (*n* = 19). Markers are plotted sparsely for clarity; error bars represent *SD*.

This imaging method thus allowed us to analyze the cAMP signal in identified D_1_ or D_2_ MSNs and we set up a protocol for transient adenylyl cyclase stimulation to analyze the onset, which mostly reflect cAMP synthesis, and the decay, which is mostly governed by PDEs activities. Because the activation of D_1_ or A_2A_ receptors induced cAMP signals that differed in amplitude and kinetics, we examined the responses to direct stimulation of AC by forskolin, thereby analyzing the cAMP signal independently of receptors. We used a fast focal application system to apply 10-s pulses of forskolin (FSK) while monitoring the cAMP response, and bath application of SKF38393 and CGS21680 at the end of the recording to identify D_1_ and D_2_ neurons (Figure 3A). For both D_1_ and D_2_ MSNs, brief forskolin stimulation resulted in a transient increase in Epac-S^H150^ signal, which could be reproduced several times with no significant change in amplitude or kinetics. These forskolin-induced cAMP transients differed between the two types of MSNs in their amplitude and decay kinetics (Figure 3B): D_1_ MSNs generated a cAMP transient that reached 67 ± 10% of *R*_max_ and had a *t*_1/2off_ of 2.5 ± 0.4 min (*n* = 112; see method for *t*_1/2off_ definition); D_2_ neurons displayed a larger cAMP signal that reached 79 ± 9% of *R*_max_ and lasted two times longer than the response in D_1_ neurons with a *t*_1/2off_ of 4.3 ± 0.8 min (*n* = 122); both amplitude and t_1/2off_ were statistically different from D_1_ (unpaired two-tailed *t*-test, *P* < 0.0001; see Table 1).

**Table 1:** **Kinetic parameters of the cAMP responses to FSK in MSNs**.

	**D1**				**D2**			
	**Amplitude (%)**	***t*_1/2off_ (min)**	***t*_max_ (s)**	***n***	**Amplitude (%)**	***t*_1/2off_ (min)**	***t*_max_ (s)**	***n***
FSK	67 ± 10	2.6 ± 0.4	52 ± 10	112	79 ± 9	4.3 ± 0.8	51 ± 8	122
FSK + SNAP	41 ± 8	2.9 ± 0.6	54 ± 9	45	62 ± 7	4.0 ± 0.5	53 ± 7	48
FSK + EHNA	72 ± 8	2.5 ± 0.6	49 ± 7	19	91 ± 5	5.4 ± 0.8	55 ± 9	19
FSK + SNAP + EHNA	66 ± 9	2.6 ± 0.7	58 ± 13	46	84 ± 6	5.2 ± 1.1	54 ± 7	44
FSK + SNAP + Bay 607,550	60 ± 12	2.0 ± 0.3	46 ± 10	38	84 ± 12	5.3 ± 2.2	59 ± 9	25

*cAMP signals were measure in wide-field imaging with Epac-S^H150^ biosensor during the application of a 10 s pulse of Forskoline (FSK, 13 μM) alone or in the presence of SNAP (100 μM), EHNA (10 μM), SNAP + EHNA and SNAP + Bay 607,550. The table shows the means (±SD) of the amplitudes, the t_max_ and t_1/2off_ in each experimental condition. n indicate the number of tested neurons*.

We thought that this difference might result from regulation of cAMP levels by PDEs, including PDE2. We observed that inhibition of PDE2 with EHNA increased the amplitude and prolonged the decay of the forskolin-induced cAMP transients exclusively in D_2_ MSNs (Figure 3C). No effect was observed in D_1_ MSNs, despite the expression of PDE2 in these neurons (Figure 3D) (Lin et al., 2010). Because PDE2 inhibition exacerbates the differences between D_1_ and D_2_ MSNs, we hypothesized that other PDE activities differently regulate the cAMP signals in these neurons. We tested this hypothesis by inhibiting most of PDE activities with IBMX (Figures 3E,F). Application of IBMX alone increased basal cAMP levels in both type of cells [28 ± 3% (*n* = 17) for D1 MSNs and 33 ± 3% (*n* = 21) for D2 MSNs], showing that adenylyl cyclase constitutively active. When most PDEs were blocked, the cAMP transient induced with 10 s FSK was larger in amplitude and its duration was considerably prolonged. The difference in kinetics between D_1_ and D_2_ was almost obliterated, demonstrating that phosphodiesterase activities differ between D_1_ and D_2_ neurons. The slope of the 10–80% onset of the cAMP transient in the presence of IBMX was measured and showed no statistical difference between D_1_ and D_2_ neurons (0.020 ± 0.005, *n* = 17 for D_1_ MSNs vs. 0.023 ± 0.007, *n* = 20 for D_2_ MSNs, expressed in ratio units per second, *p* = 0.199). This suggests that the rate of cAMP synthesis is similar in both cell types. Further work is needed to identify the specific contribution of each PDEs in determining the shape of transient cAMP signals in D_1_ and D_2_ MSNs.

### PDE2 activation by cGMP limits cAMP accumulation in both D_1_ and D_2_ MSNs

**Figure 4:**
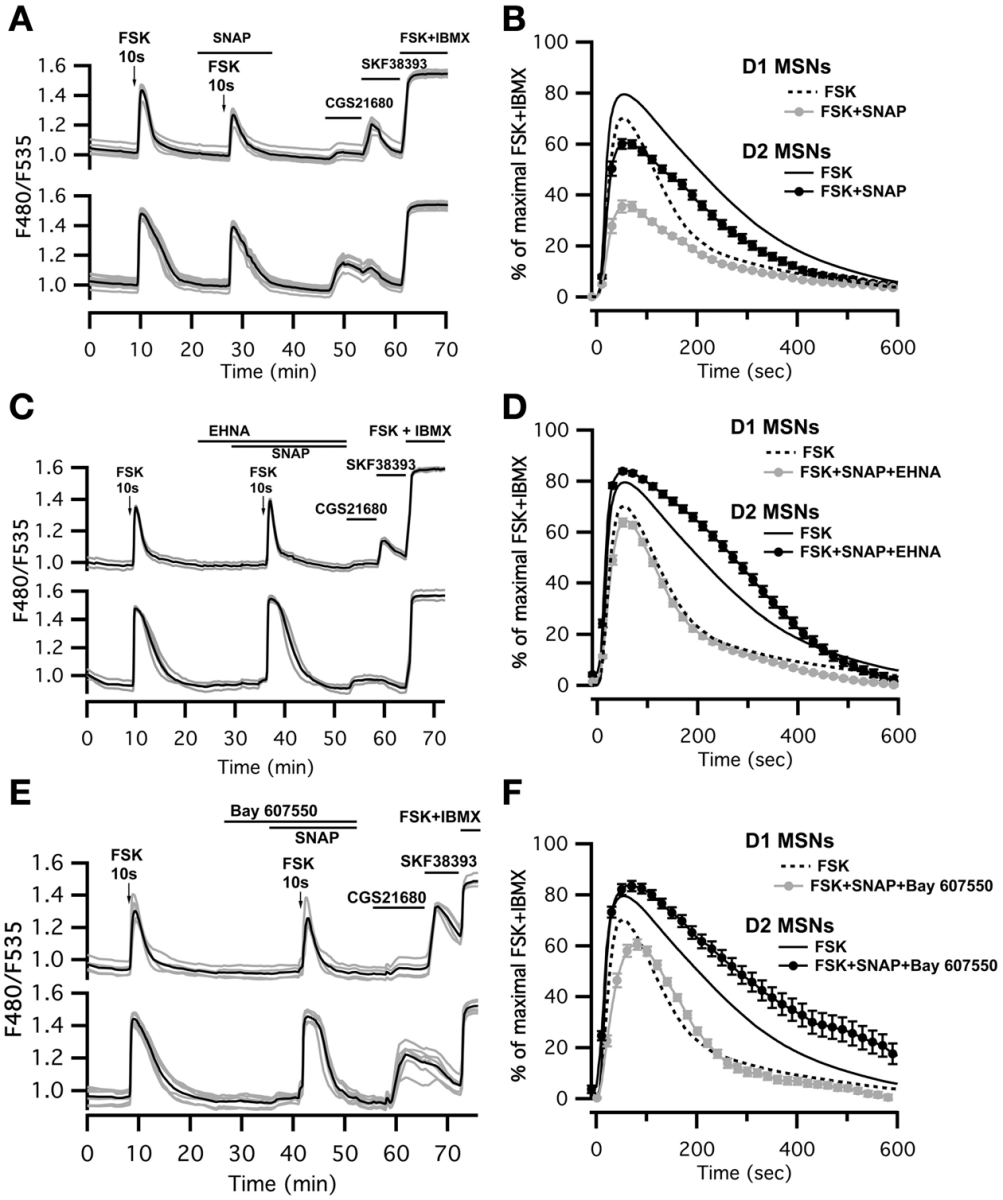
**SNAP reduces the cAMP/PKA signal in both D_1_ and D_2_ MSNs.** cAMP signals were measure in wide-field imaging with Epac-S^H150^ biosensor during the application of a 10 s pulse of Forskolin (FSK, 13 μM). **(A)** Activation of soluble guanylyl cyclase with SNAP (100 μM) strongly reduced the amplitude of the cAMP response induced by brief (10 s) FSK application in both D_1_ and D_2_ MSNs. Inhibition of PDE2 with EHNA (10 μM) **(C)** or Bay 607,550 (100 nM) **(E)** abolished the effect of SNAP on the cAMP response. **(B)** Mean responses in D_1_ (*n* = 45) and D_2_ (*n* = 48) MSNs to brief FSK stimulation in the presence of SNAP. **(D)** Mean responses in D_1_ (*n* = 46) and D_2_ (*n* = 44) MSNs to brief FSK stimulation in the presence of SNAP plus EHNA; **(F)** Mean responses in D_1_ (*n* = 38) and D_2_ (*n* = 25) MSNs to brief FSK stimulation in the presence of SNAP plus Bay 607,550. **(B,D,F)** dashed and solid lines represent the average response to brief FSK alone in D_1_ and D_2_ MSNs, respectively. Markers are plotted sparsely for clarity; error bars represent *SD*.

We then tested whether increasing PDE2 activity with cGMP affected cAMP signaling in MSNs: we compared the cAMP transients in the absence and in the presence of the NO donor SNAP (100 μM). Application of SNAP in the bath had no effect *per se* on basal cAMP signals (Figure 4A). However, the cAMP transient was reduced in amplitude by about 20–30% in both striatonigral and striatopallidal MSNs (Figure 4B; Table 1). The decay time-course, however, did not change significantly from the respective control (Figure 4B; Table 1).

This negative control exerted by NO was abolished when the PDE2 inhibitor EHNA (10 μM) was applied simultaneously with the NO donor SNAP (100 μM) (Figures 4C,D; Table 1). The application of EHNA alone or in the presence of SNAP had no effect on basal cAMP signal (Figure 4C). Since EHNA also inhibits adenosine deaminase, we also tested the specific PDE2 inhibitor BAY60-7550 at 100 nM (Figures 4E,F): this drug also blocked the inhibitory effect of NO donors on cAMP transients. These results show that the inhibitory crosstalk exerted by cGMP on cAMP transients is mediated by PDE2.

### The NO/cGMP/PDE2 pathway limits PKA activation upon a sub-second dopamine signal

**Figure 5:**
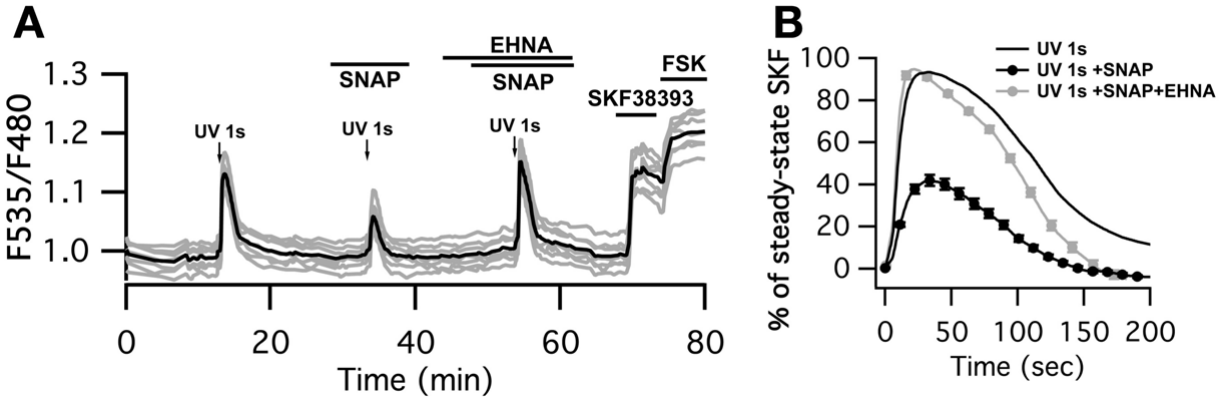
**SNAP inhibits the PKA response to brief (1 s) dopamine uncaging in D_1_ MSNs. (A)** MSNs expressing AKAR3 were imaged in wide-field microscopy to measure the PKA response to dopamine. Arrows indicate the UV flash. Flash photolysis of caged dopamine (NPEC-dopamine, 5 μM in the bath) induced a transient ratio increase. SNAP decreased this response, and the PDE2 inhibitor EHNA (10 μM) restored the response to caged dopamine to its initial level. **(B)** Mean responses in D_1_ to 1s UV flash alone (*n* = 84), in the presence of SNAP (*n* = 80) and SNAP + EHNA (*n* = 74). For each flash, responses were normalized with respect to the steady-state response to SKF38393. Markers are plotted sparsely for clarity; error bars represent *SD*.

Finally, we checked whether the negative regulation exerted by cGMP on the cAMP signal was sufficient to down-regulate PKA activity. Physiological activation of D_1_-like receptors is associated with phasic dopamine release related to a reward or novelty (Garris and Wightman, 1994; Gonon, 1997; Schultz and Dickinson, 2000). We mimicked phasic dopamine signals by uncaging NPEC-dopamine with a flash of UV light of 1 s duration while monitoring PKA activity with AKAR3. At the end of the experiment, bath application of SKF38393 allowed for the identification of D_1_ neurons (Figure 5A). D_2_ neurons, which did not respond to dopamine uncaging or SKF38393, were ignored. As already described (Castro et al., 2013), D_1_ MSNs displayed a large and transient response to 1 s dopamine uncaging that reached 90 ± 2% *n* = 84 of the maximal steady-state response to SKF38393 response (Figure 5B). In the presence of the NO donor SNAP, the amplitude of the PKA signal was decreased by 45% (42 ± 3% *n* = 80). PDE2 inhibition by 10 μM EHNA reverted the inhibitory effect of SNAP (93 ± 4% *n* = 74) showing that PDE2 is indeed the mediator of this inhibitory effect.

Interestingly, the inhibitory effect of SNAP appeared larger at the level of PKA than at the level of cAMP, showing that a moderate change in cAMP dynamics is amplified at the PKA level.

## Discussion

Previous studies have clearly highlighted NO/cGMP as a key player in detecting and reinforcing corticostriatal glutamatergic and dopaminergic neurotransmission (Calabresi et al., 2007; West and Tseng, 2011). In this report, we highlight the importance of the post-synaptic level where NO affects the integrative properties of MSNs. Our data demonstrate the functional importance of PDE2 as a target of the NO/cGMP pathway, and its importance in the regulation of transient cAMP responses. With the high resolution provided by our improved biosensor, we also highlight significant differences between D_1_ and D_2_ MSNs in their response to transient AC stimulation, a difference that results from differences in phosphodiesterase activities.

### PDE2 as a major target of cGMP in the striatum

The physiological effects of the NO/cGMP pathway largely depend on its targets which include cGMP-gated ion channels, cGMP-dependent kinase protein (PKG) and cGMP-regulated PDEs. Interestingly, brain regions such as the hippocampus and the striatum which express high levels of sGC (Matsuoka et al., 1992; Ding et al., 2004), do not express much PKG protein (el-Husseini et al., 1995; El-Husseini et al., 1999) but very high levels of PDE2 (Repaske et al., 1993; Van Staveren et al., 2003). This suggests that the major role of cGMP may be to regulate cAMP levels via PDE2 activation, rather than to directly activate PKG. This hypothesis is clearly supported by our experiments which show that one major functional effect of NO-triggered cGMP production is the activation of PDE2 which then strongly reduce the cAMP response in both D_1_ and D_2_ neurons.

The inhibitory effect of NO on the transient response was much more pronounced when considering the effect at the PKA level than at the cAMP level. This results appears counterintuitive as one might expect PKA to smooth-out small variations in cAMP concentration. This non-linearity in signal transduction from cAMP level to PKA level may result from positive feedback controls such as that mediated by DARPP-32: DARPP-32 was shown to prolong the PKA response once it was activated (Le Novere et al., 2008; Castro et al., 2013); in our experiments, a cAMP response that has been lowered by the NO-cGMP pathway may be insufficient to phosphorylate DARPP-32, while a larger cAMP signal may phosphorylate more DARPP-32, therefore inhibit Phosphatase 1 more efficiently and thus potentiate the net PKA effect. Such non-linear effects certainly determine the integrative properties of striatal neurons and further modeling studies are needed to understand how this parameter comes into play in the current learning theories.

### Dichotomous cAMP responses in MSNs

While the characteristic segregation of MSNs in two different cell types is notoriously difficult to study on dissociated neurons, biosensor imaging provides a measurement at the level of individual mature neurons which can be identified on the basis of their response to an agonist of either dopamine D_1_ or adenosine A_2A_ receptor (Castro et al., 2013). In this study, the ^T^Epac^VV^ cAMP sensor was improved to increase its affinity for cAMP and decrease it size, yielding a biosensor which proved highly efficient to monitor cAMP signals in striatal neurons. This new biosensor revealed the larger and desensitizing profile of the D_1_ response which differed from the smaller and steady A_2A_ response in D_2_ MSNs. These differences may be related to different regulations at the level of receptor transduction and remain to be explored in more details.

Our experiments also revealed a clear differences between these two types of MSNs that lie downstream of the receptors, which appeared when AC was transiently stimulated with forskolin: the cAMP signals were stronger and lasted longer in D2 MSNs than in D1 neurons. The AC activities do not explain the differences observed in these cells because the rate of cAMP production upon forskolin stimulation was similar in D1 and D2 MSNs as indicated by a similar onset slope. This dichotomy depends on differences in PDE activity since the broad-spectrum PDE inhibitor, IBMX, suppressed the differences between MSNs. Specific inhibition of PDE2 increased the cAMP signal exclusively in D2 neurons, despite the expression of PDE2 in both D1 and D2 neurons (Lin et al., 2010): this suggests that PDE2 is at the forefront in the degradation of cAMP in D2 neurons. Transient cAMP responses recover faster in D1 neurons than in D2 neurons, and this recovery is not affected by PDE2 inhibition suggesting that another PDE takes over PDE2 in D1 neurons. Based on these results, we consider that amplitude and decay of the cAMP signal are governed by a complex and non-linear network, which may include subcellular compartmentation, in which PDE2 appears as one critical element. Which PDE (or combination) is at the forefront in D1 neurons remains to be determined, possibly PDE1A (Polli and Kincaid, 1994) and/or PDE10A (Xie et al., 2006).

### NO increases cGMP levels in both types of MSNs

This clear-cut segregation of MSNs into two populations with different pharmacological profiles and PDE activities that was observed for the cAMP/PKA signaling cascade was not seen at the level of cGMP with the cGMP biosensor Cygnet: sGC stimulation with NO donors produced a large and homogeneous increase in cGMP levels in all MSNs, which is consistent with the high levels of sGC present in both neuron types (Ding et al., 2004).

A tonic production of cGMP in response to the spontaneous release of NO was reported in the hippocampus, optic nerve, thalamus (Garthwaite et al., 2006; Hopper and Garthwaite, 2006; Hepp et al., 2007), as well as in the striatum (Calabresi et al., 1999; Galati et al., 2008). Our experiments in MSNs contrast with these observations as no changes in cGMP concentration was obtained upon bath application of ODQ or IBMX (Figure 1). However, data in the striatum were obtained in quite different experimental conditions: either the glutamatergic corticostriatal afferent fibers were repeatedly stimulated which should activate NOS interneurons (Calabresi et al., 1999), or the recordings were performed *in vivo* where a number of other parameters can directly or indirectly contribute to a tonic activation of these NO-synthesizing cells (West and Grace, 2000, 2004; Galati et al., 2008). This suggests that, *in vivo*, NOergic interneurons tonically release NO which downregulate the dopamine-induced PKA response of MSNs. Integrating this cellular mechanism into the whole animal would be of paramount importance to elucidate the clinical influence of compounds interfering with NO/cGMP pathway in disabling disorders associated with the striatum.

## Author contributions

Liliana R. V. Castro and Pierre Vincent contributed equally to this work (co-last authors). Danièle Paupardin-Tritsch, Kees Jalink, Pierre Vincent, and Liliana R. V. Castro supervised the project. Liliana R. V. Castro, Marina Polito, and Jeffrey Klarenbeek performed the experiments. Liliana R. V. Castro, Marina Polito, Danièle Paupardin-Tritsch, and Pierre Vincent wrote the manuscript.

### Conflict of interest statement

The authors declare that the research was conducted in the absence of any commercial or financial relationships that could be construed as a potential conflict of interest.
